# Supplementary material for: Deciphering Ca2+ permeation and valence selectivity in CaV1: Molecular dynamics simulations reveal the three-ion knock-on mechanism
Source: Proc Natl Acad Sci U S A. 2025 May 29;122(22):e2424694122. doi: 10.1073/pnas.2424694122 (PMC12146731; doi:10.1073/pnas.2424694122)
Supplement: Supplementary file 1 — Appendix 01 (PDF) [file pnas.2424694122.sapp.pdf]

Supporting Information for

Deciphering Ca^{2+} Permeation and Valence Selectivity in Ca_V1: Molecular Dynamics Simulations Reveal the Three-Ion Knock-on Mechanism

Lingfeng Xue, Nieng Yan and Chen Song

Chen Song.
E-mail: c.song@pku.edu.cn

This PDF file includes:

Supporting text
Figs. S1 to S5
Tables S1 to S3
Legends for Movies S1 to S6
SI References

Other supporting materials for this manuscript include the following:

Movies S1 to S6

Supporting Information Text

Conductance calculation with ECC. Here we detail the formula for the conductance calculation in MD simulations with ECC, and validate it with the equilibrium fluctuation method.

The formula used in the manuscript is:

$$g = \frac{I}{V} = \frac{Nq}{tEL_z}\frac{1}{f_{\mathrm{ECC}}} \qquad [1]$$

First, we note the conceptual differences between the ECC method and the naive charge scaling method. The ECC method is used to fine-tune the relative permittivity to optimize the electrostatic interactions by scaling down the charges. The purpose is to obtain the "correct" dynamic behavior of ions to match the experimental observations in our case. Therefore, the ions in the ECC simulations actually represent the "experimental" or "real" ions. Consequently, when the conductance is calculated, the charge should not be scaled. In 2020, Předota and Biriukov provided a comprehensive discussion on this matter (1).

The above formula is different from the conventional method, with an additional scaling factor f_{ECC}. The reason is as follows: In the ECC method, theoretically, the Coulomb interactions between charged particles are scaled, but the charges of charged particles should remain unchanged. Therefore, the forces on charged particles caused by the external electric field or transmembrane potential should remain unchanged. However, in the ECC MD simulations, the electric forces sensed by the ions are qEf_{ECC} instead of qE, where E is the input electric field in the MD simulations. Therefore, in conductance calculation, we should use the scaled effective electric field Ef_{ECC} rather than the input electric field E. This has also been discussed by Předota and Biriukov from another perspective: "To run a simulation at E by scaled-up input field E/f_{ECC} and comparing with results of experiment or ab initio or polarizable model at E is more appropriate" (1). Note that in their discussion, E is the effective (experimental) electric field, while E/f_{ECC} is the input electric field. So basically, $E_{\mathrm{eff}} = E_{\mathrm{input}}f_{\mathrm{ECC}}$, and one should use E_{eff} to calculate the conductance, hence the scaling factor f_{ECC} is introduced in the above formula.

Apart from the relative permitivity, nothing is changed in the ECC simulations, so we believe the classic way of calculating the membrane potential from the applied constant electric field is still applicable (2), as long as the effective electric field is used. Then the formula for calculating conductance should be:

$$g = \frac{I}{V_{\mathrm{eff}}} = \frac{Nq}{tE_{\mathrm{eff}}L_z} = \frac{Nq}{tE_{\mathrm{input}}L_z}\frac{1}{f_{\mathrm{ECC}}} \qquad [2]$$

Further validation

To further validate the above consideration, we simulated a 0.1 M KCl and a 0.1 M $CaCl_2$ solution with a scaling factor of 0.5. Three methods were used to calculate the conductance of cations: 1) equilibrium fluctuation method; 2) apply an electric field and calculate the conductance with the scaled electric field; 3) apply an electric field and calculate the conductance with the raw input electric field. We performed 100 ns simulations with 5 replicates for each case.

The fluctuation method gives the conductance as (3)

$$g_{\mathrm{fluc}} = \frac{1}{k_{\mathrm{B}}T}\frac{\langle|\Delta Q(t)|^2\rangle}{2t} = \frac{q^2}{k_{\mathrm{B}}T}\frac{\langle n(t)^2\rangle}{2t} \qquad [3]$$

where q is the charge of ion, k_{B} is the Boltzmann constant, T is the simulation temperature, t is the simulation time, $n(t) = \frac{\Delta z(t)}{L_z}$ is the number of ions permeated in z-direction, calculated as the displacement in z-direction Δz and the z-direction box size L_z, and $\langle n(t)^2\rangle$ is the fluctuation of $n(t)$. Each simulation trajectory was spliced into 10 segments to calculate the conductance.

In the electric field method with scaling correction, conductance is

$$g_{\mathrm{E,corr}} = \frac{1}{f_{\mathrm{ECC}}}\frac{\langle n(t)\rangle q}{tEL_z} \qquad [4]$$

while for electric field method without scaling correction, conductance is

$$g_{\mathrm{E,nocorr}} = \frac{\langle n(t)\rangle q}{tEL_z} \qquad [5]$$

where E is the applied electric field.

As shown in Fig. S5, the conductance calculated from the corrected electric field ($g_{\mathrm{E,corr}}$) is consistent with the fluctuation method (g_{fluc}), while the conductance from the raw input electric field ($g_{\mathrm{E,nocorr}}$) underestimates the conductance by a factor of 0.5. These results demonstrate that the conductance should be calculated with the corrected electric field by the scaling factor.

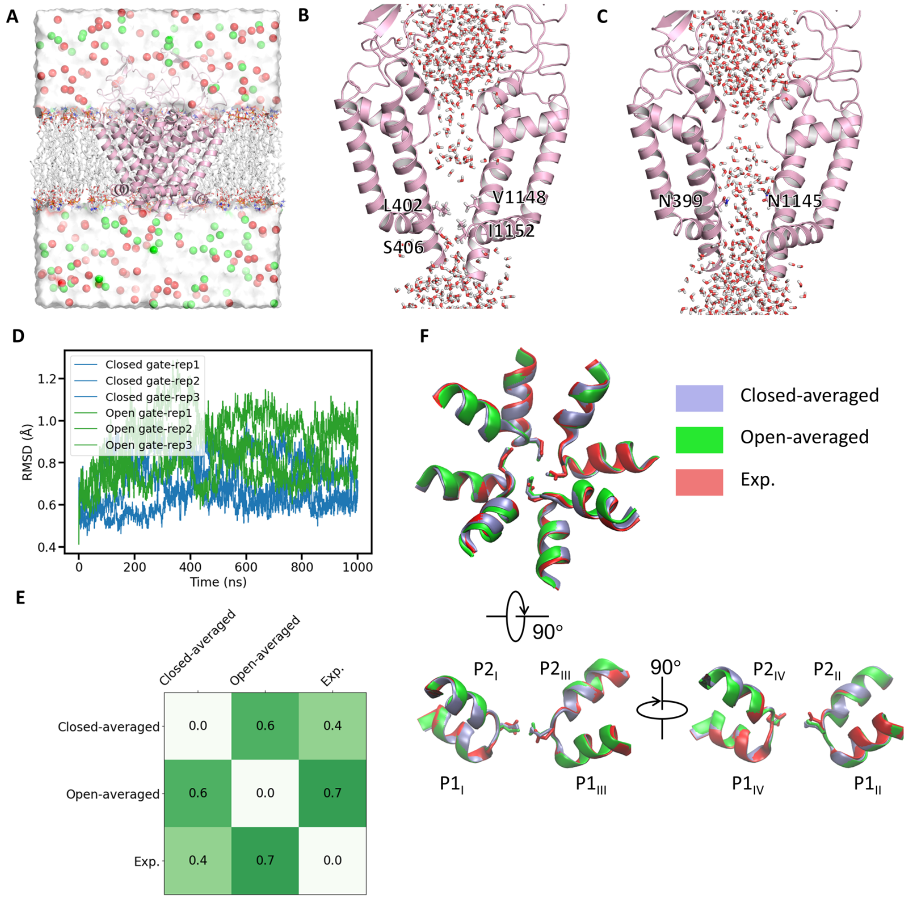

Fig. S1. Modeling the open-state structure of $Ca_V1.3$. (A) System overview. The $Ca_V1.3$ protein is shown with cartoon. POPC lipids are shown with sticks. Water is shown as white surface. Calcium and chloride atoms are shown in green and red spheres. (B) Side view of the channel with a closed gate. The $Ca_V1.3$ protein is shown with cartoon, with repeat II and IV removed for clarity. Side chains of key residues around the intracellular gate are shown as sticks, with residue numbers labeled. Water molecules around the pore axis are shown as sticks as well. (C) Same as (A), but after adding restraints to the S6 gate region according to the open-state Na_V structure. The gate region is hydrated after dilation. (D) RMSDs of SF backbone of closed state and open state in MD simulations, with experimental SF structure (PDB ID: 7UHG) as reference. Position restraints were applied to gate region only, and three replicate simuations were conducted for each condition. (E) RMSDs between SF backbone structures of the averaged closed state, averaged open state and the experimental structure (unit: Å). The average structures were calculated with the latter 500 ns of the three trajectories for each case. (F) Superimposition of the three SF structures of (E).

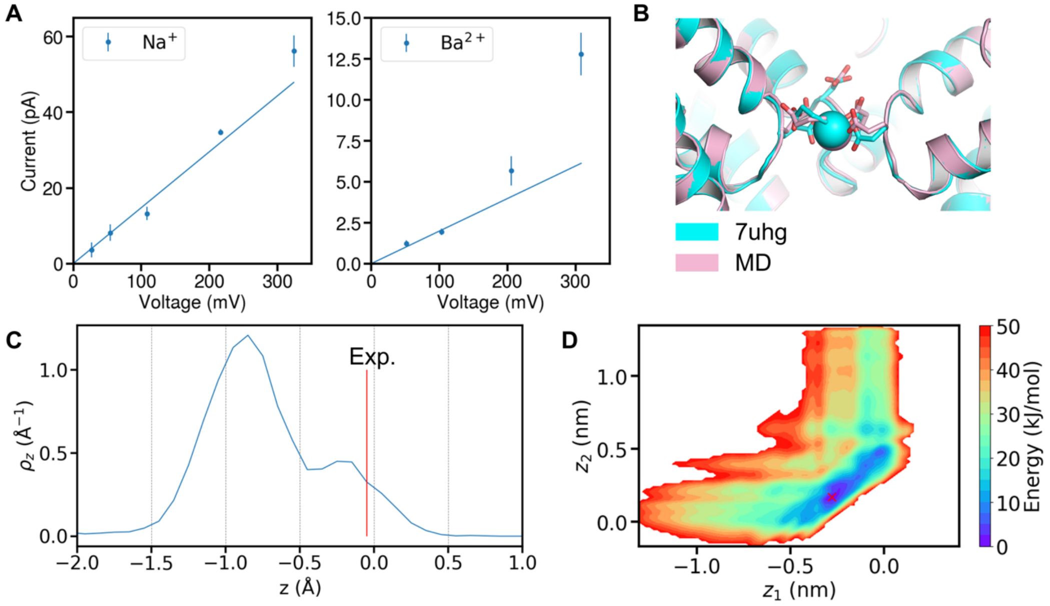

Fig. S2. Further validation of the simulation system and protocol by electrophysiological and structural features. (A) The current-voltage relationship for Na^{+} and Ba^{2+}. The blue lines represent the results from linear fitting through the origin. (B) The representative SF configuration in one-Ca^{2+} simulations (pink) compared with the experimental structure (cyan) (PDB: 7UHG). The two structures are aligned by Cα atoms of SF. (C) The z-distribution of Ca^{2+} in MD simulations, with the red line representing the experimental density peak. The reference position is the center of the EEEE locus. (D) Two-Ca^{2+} PMF from Metadynamics simulations, with the red cross indicating the most stable configuration.

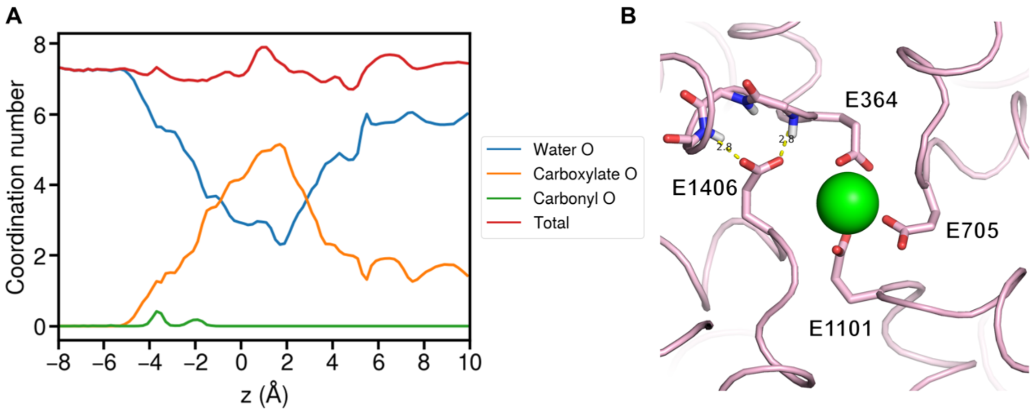

Fig. S3. Coordination interactions with permeating Ca^{2+} ions in ion permeation simulations. (A) The coordination number distribution of Ca^{2+} with oxygen atoms along the z-axis. The blue, orange, green and red lines represent the coordination with water oxygens, carboxylate oxygens, carbonyl oxygens, and all oxygens, respectively. (B) Top view of the asymmetric coordination of the EEEE locus. The protein structure is shown as ribbons, with the side chains of the EEEE locus shown as sticks. The green sphere represents the bound Ca^{2+}. The hydrogen bonds formed between the backbone nitrogens and the E1406 residue are indicated.

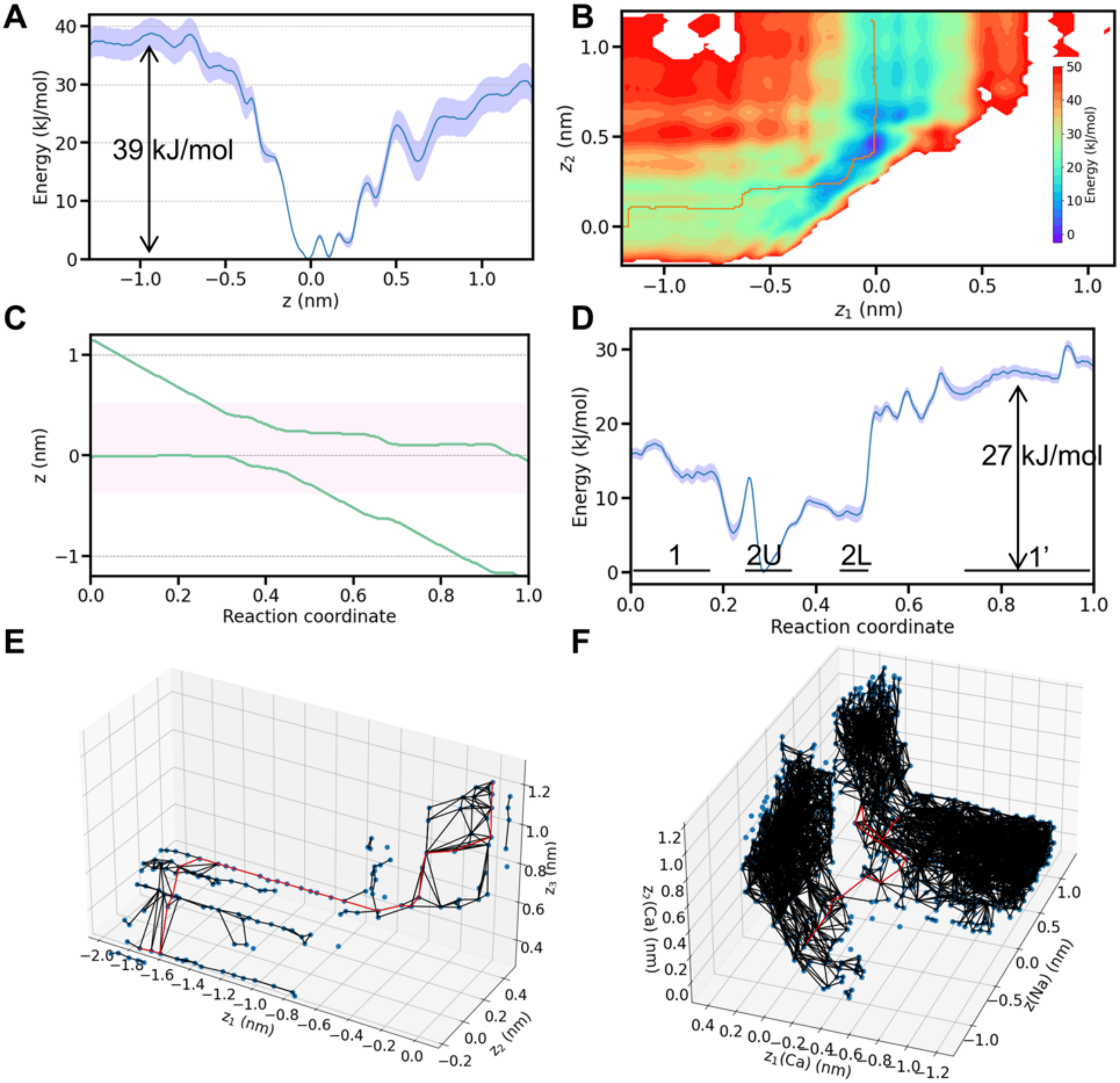

Fig. S4. PMF results for ion permeation and selectivity. (A) One-Ca^{2+} PMF profile, with the blue shaded area representing error bars. The arrow indicates the energy barrier for Ca^{2+} permeation. (B) Two-Ca^{2+} PMF results, with the minimum free energy path (MFEP) indicated as the orange line. (C) The coordinated motion of two Ca^{2+} ions along the z-axis of the MFEP. The pink shaded area indicates the SF region. (D) The free energy profile along the MFEP of the two-Ca^{2+} PMF, with labeled states according to positions in (C) and the blue shaded area representing error bars. The arrow indicates the energy barrier for Ca^{2+} permeation. (E) Three-Ca^{2+} PMF results with an energy cutoff of 16 kJ/mol. The local energy minima are shown as blue nodes, with the connecting paths shown as black edges. The red line represents the MFEP. (F) Similar to (E), but for one-Na^{+}-two-Ca^{2+} PMF with a cutoff of 36 kJ/mol.

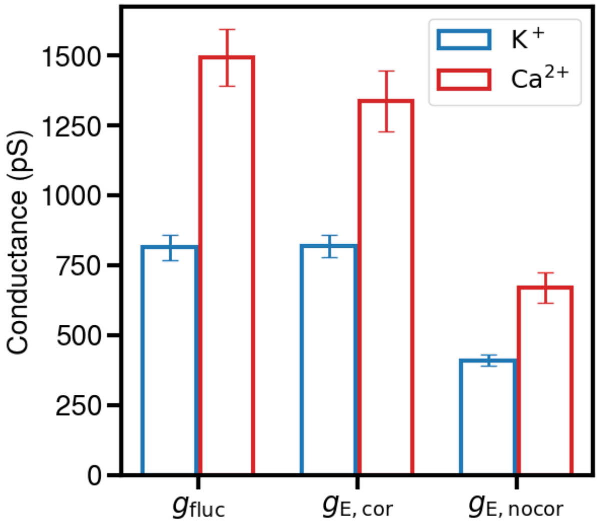

Fig. S5. Comparison between conductances obtained with different methods: fluctuation method without applied electric field ($g_{\rm fluc}$), applied electric field method with scaling correction ($g_{\rm E,corr}$), applied electric field method without scaling correction ($g_{\rm E,nocorr}$).

Table S1. Conventional MD simulations summary.

Ionic condition	Electric field (V/nm)	f_{ECC}	Mutant	Time (µs)	Replicate number
single Ca + 0.15 M Na*	0	0.87	WT	0.5	1
0.15 M Ca	0.01	0.82	WT	1	3
0.15 M Ca	0.01	0.87	WT	1	3
0.15 M Ca†	0.01	0.87	WT	2	5
0.15 M Ca	0.01	0.89	WT	1	3
0.15 M Ca	0.02	0.87	WT	1	3
0.15 M Ca	0.03	0.87	WT	1	3
0.15 M Na	0.0025	0.87	WT	0.5	3
0.15 M Na	0.005	0.87	WT	0.5	3
0.15 M Na	0.01	0.87	WT	0.5	3
0.15 M Na	0.02	0.87	WT	0.3	3
0.15 M Na	0.03	0.87	WT	0.3	3
0.15 M Ba	0.005	0.87	WT	2	3
0.15 M Ba	0.01	0.87	WT	2	3
0.15 M Ba	0.02	0.87	WT	1	3
0.15 M Ba	0.03	0.87	WT	0.5	3
0.15 M Ca + 0.15 M Na	0.01	0.87	WT	2.5	15
0.15 M Ca	0.03	0.87	E364A	1	3
0.15 M Ca	0.03	0.87	E705A	1	3
0.15 M Ca	0.03	0.87	E1101A	1	3
0.15 M Ca	0.03	0.87	E1406A	1	3
0.15 M Ca	0.03	0.87	D706A	1	3
0.15 M Ba	0.03	0.87	E364A	0.5	3
0.15 M Ba	0.03	0.87	E705A	0.5	3
0.15 M Ba	0.03	0.87	E1101A	0.5	3
0.15 M Ba	0.03	0.87	E1406A	0.5	3
0.15 M Ba	0.03	0.87	D706A	0.5	3
0.15 M Ca	0.01	0.87	WT	1	3
0.15 M Ca‡	0.01	0.87	WT	1	3
0.15 M Ca + 0.15 M Na	0.02	0.87	WT	2	5
Total simulation time: 124.3 µs					

*With closed gate structure.
†Temperature 300 K.
‡With closed gate structure.

Table S2. Metadynamics simulations summary.

Condition	Time (µs)	Replicate number	Gate state
One Ca	4	1	Open
Two Ca	4	1	Open
Two Ca	4	1	Closed
Three Ca	6	1	Open
One Na + two Ca	6	1	Open
Total simulation time: 24 µs			

Table S3. Key residues of $Ca_V1.3$ in conventional and generic residue numbering system.

Residue	$Ca_V1.3$ residue number	Generic residue numbering system
E	364	P2I,28
E	705	P2II,28
E	1101	P2III,28
E	1406	P2IV,28
D	706	P2II,29
D	368	P2I,32
N	399	S6I,30
N	745	S6II,30
N	1145	S6III,30
N	1457	S6IV,30

Movie S1. A Ca^{2+} permeation trajectory through Ca_V1 in pure-Ca^{2+} condition.

Movie S2. A Ca^{2+} permeation trajectory through Ca_V1 in bi-cation condition.

Movie S3. A Na^{+} permeation trajectory through Ca_V1 in pure-Na^{+} condition.

Movie S4. The first Na^{+} permeation trajectory through Ca_V1 in bi-cation condition.

Movie S5. The second Na^{+} permeation trajectory through Ca_V1 in bi-cation condition.

Movie S6. The third Na^{+} permeation trajectory through Ca_V1 in bi-cation condition, in which two Na^{+} translocated together.

References

1. M Předota, D Biriukov, Electronic continuum correction without scaled charges. *J. Mol. Liq.* **314**, 113571 (2020).
2. B Roux, The membrane potential and its representation by a constant electric field in computer simulations. *Biophys. J.* **95**, 4205–4216 (2008).
3. B Roux, T Allen, S Berneche, W Im, Theoretical and computational models of biological ion channels. *Q. Rev. Biophys.* **37**, 15–103 (2004).
